# Environmental distribution of prokaryotic taxa

**DOI:** 10.1186/1471-2180-10-85

**Published:** 2010-03-22

**Authors:** Javier Tamames, Juan José Abellán, Miguel Pignatelli, Antonio Camacho, Andrés Moya

**Affiliations:** 1 Unidad Mixta de Investigación en Genómica y Salud, Centro Superior de Investigación en Salud Pública (CSISP) y Universidad de Valencia (Instituto Cavanilles de Biodiversidad y Biología Evolutiva). Avenida de Cataluña 21, 46020 Valencia, Spain; 2 CIBER en Epidemiología y Salud Pública (CIBERESP), Spain; 3 Instituto Cavanilles de Biodiversidad y Biología Evolutiva y Departamento de Microbiología y Ecología Universidad de Valencia. C/Dr. Moliner 50, 46100 Burjassot, Valencia, Spain; 4 Centro Nacional de Biotecnología (CNB-CSIC). C/Darwin 3, 28049 Madrid, Spain

## Abstract

**Background:**

The increasing availability of gene sequences of prokaryotic species in samples extracted from all kind of locations allows addressing the study of the influence of environmental patterns in prokaryotic biodiversity. We present a comprehensive study to address the potential existence of environmental preferences of prokaryotic taxa and the commonness of the specialist and generalist strategies. We also assessed the most significant environmental factors shaping the environmental distribution of taxa.

**Results:**

We used 16S rDNA sequences from 3,502 sampling experiments in natural and artificial sources. These sequences were taxonomically assigned, and the corresponding samples were also classified into a hierarchical classification of environments. We used several statistical methods to analyze the environmental distribution of taxa. Our results indicate that environmental specificity is not very common at the higher taxonomic levels (phylum to family), but emerges at lower taxonomic levels (genus and species). The most selective environmental characteristics are those of animal tissues and thermal locations. Salinity is another very important factor for constraining prokaryotic diversity. On the other hand, soil and freshwater habitats are the less restrictive environments, harboring the largest number of prokaryotic taxa. All information on taxa, samples and environments is provided at the envDB online database, http://metagenomics.uv.es/envDB.

**Conclusions:**

This is, as far as we know, the most comprehensive assessment of the distribution and diversity of prokaryotic taxa and their associations with different environments. Our data indicate that we are still far from characterizing prokaryotic diversity in any environment, except, perhaps, for human tissues such as the oral cavity and the vagina.

## Background

The patterns of species distribution and diversity, which are fairly well-known for macroorganisms, are not altogether understood for microorganisms. Some ecological trends that have already been observed for macroorganisms, such as taxa-area or distance-decay relationships [1], and especially the existence of biogeographical patterns, have been proposed to possibly exist also for microorganisms, thus pointing to the existence of common, global rules that govern the ecology of all living forms. Some analyses support the ubiquity of several prokaryotic species [2,3], but also the apparent existence of biogeographic patterns for some others [3-7].

The study of ecological trends in microorganisms has been traditionally hampered by different factors. First, the methods used to catalogue microbial diversity (mostly based on sequencing the 16S rDNA gene) are expensive, time-consuming, biased and inadequate for massive screening, although technologic advances in DNA sequencing technology can change this picture dramatically [8-10]. Another serious problem is the lack of a proper concept of prokaryote species. The current definition is mainly based on genotypic characteristics, such as the percentage of DNA-DNA hybridization or the percentage of identity between the 16S rDNA molecules [11]. However, this approach is known to group rather different strains together which should probably be considered as different species (as in *Escherichia coli*), or to separate organisms with an almost identical gene complement (as in the genus *Bacillus*). The ongoing debate on this topic includes the proposal that similarity in lifestyle, and not just in genes, is the best approach to classify microorganisms [12,13]. Similar ecological and metabolic features are scattered through different clades among the prokaryotic world, conforming specific metabolic groups of prokaryotes, such as the different metabolic types of sulfur bacteria [14]. Polyphasic approaches [15], including an overview on genotypic, phenotypic, and ecological features, would be necessary to better understand the global distribution of prokaryotes. But in practice, most studies simply use the so-called Operational Taxonomic Units (OTUs) [16] obtained, for instance, by grouping 16rDNA genes at the 97-98% threshold of identity, as a way to circumvent the absence of an adequate definition of species [17].

Also the massive number of existing species makes cataloguing microbial diversity difficult [18]. Most sampling efforts miss present species, which, in some cases, can produce an inadequate picture of the patterns that underlie community structure [1].

Furthermore, knowledge about the most determining factors that shape the distribution of bacteria in the different environments is still limited. It is quite usual to ascribe whole bacterial clades to a single environment by identifying them as for instance, marine or terrestrial. Should we make this simple categorization? Could this not be a consequence of our incomplete and biased knowledge of the different environments and taxa? To study ecological trends, it is important to count on an accurate description of habitats and, accordingly, it would be most helpful to adopt standardized descriptions for the environmental characteristics of the samples. An interesting initiative in this direction is being carried out with the development of the MIGS/MIMS (minimum information about a genomic/metagenomic sequence) specifications by the Genomic Standards Consortium [19].

Nowadays, however, there are a big number of studies inspecting the presence of particular taxa in different environments. The analysis of the presence of taxa in different environments for which many samples are available is a valuable approach to in part overcome some of the limitations pointed above. The use of these data may allow to obtaining conclusions on how environmental features and taxa-specific properties influence the patterns of microbial distribution.

In this study, we present a comprehensive analysis of the relationships between individual prokaryotic taxa and different environments, in an attempt to cover two main objectives: firstly, to describe the environmental distribution of taxa, in order to explore the existence of environmental preferences for taxa and the commonness of specialists (environment-specific species) and generalists (ubiquitous, cosmopolitan species), at different taxonomic levels (from phyla to species); second, to describe environmental variation according to taxa distribution in an attempt to ascertain common features between different environments and to determine the most significant environmental characteristics. In both cases, we show the most remarkable trends that could orientate future studies on these issues. Although partially similar studies were performed in the past [20], this is, as far as we know, the most comprehensive assessment of the environmental distribution and diversity of prokaryotic taxa.

## Results

Previous references have attempted to characterize the patterns of distribution and diversity of some taxa by proposing, for instance, the existence of environment-specific taxa, or even whole clades [5,21]. But some of these results may have been greatly influenced by the coarse-grained resolution of the environmental classification used, especially by a limited number of samples which can obscure the real patterns of taxonomic distribution and diversity. To obtain results that are as accurate and complete as possible, we used the complete set of environmental samplings stored in the GenBank database, each of which contains a variable number of 16S rDNA sequences found at the corresponding locations. This set of environmental data is probably the richest available source of information on the distribution of prokaryotic organisms and, to our knowledge, has not been used as a whole before. By exploring a high number of samples from a given environment, we expect to increase the statistical power to detect patterns in sequence diversity for that environment.

It is sensible to think that patterns of ecological and metabolic diversity may depend on the specific level of taxonomic and environmental resolution. In this study we have considered all possible taxonomic ranks, from phyla to species, in order to explore how the trends change with taxonomic resolution (in some instances, the results are detailed and discussed for the family taxonomic rank). Likewise, we have created a novel classification of environments composed of three nested levels of environment classes with increasing resolutions (Table 1). Each sample is classified using this scheme. The sequences from the samples have been grouped into OTUs using a threshold of 97% identity, and have been taxonomically classified at the deepest possible level. Because we can identify the taxa present in each of the environmentally classified samples, we can address the study of the relationships between taxa and environments.

**Table 1:** Classification of environments

Supertype	Type	Subtype	Samples	OTUs	Seqs
		Coastal waters	65	3620	8596
		
		Open waters	159	5087	13088
		
	Saline waters (300)	Deep waters	34	1752	3621
		
		Lakes	23	727	973
		
		Other	19	964	1452
	
	Saline sediment (199)		199	8514	14300
	
		Aquifers	42	1606	2087
		
Aquatic (127)		Groundwaters	47	1768	3212
		
	Freshwaters (501)	Lakes	131	4326	8505
		
		Rivers	67	2823	5467
		
		Drinking waters	14	504	983
		
		Wastewaters	200	5659	9139
	
	Freshwater sediment (101)		101	4279	6670
	
	Freshwaters-Saline waters interfase (31)		31	1047	1835
	
	Marine host-associated (145)		145	5116	8029

		Agricultural	110	8324	18987
		
		Arctic	59	4186	6749
		
		Arid	30	1344	1738
		
		Cave	21	682	1010
		
	Soil (584)	Forest	63	4980	7880
		
Terrestrial (732)		Grassland	14	4910	5860
		
		Rocks	67	2920	4039
		
		Saline	27	1365	2859
		
		Other	193	10360	17297
	
	Plants (148)	Rhizosphere	100	4779	7664
		
		Other	48	1888	3741

Thermal (190)	Hydrothermal (79)		79	2981	5077
	
	Geothermal (111)		111	2705	6027

	Animal host (52)		52	1292	2661
	
		Human	87	9715	54725
		
		Cattle	73	3418	6519
		
	Gastrointestinal tract (331)	Mouse	19	3582	18330
		
Host-associated (463)		Insect	79	3545	8838
		
		Other	73	2384	4556
	
	Oral (39)		39	886	10546
	
	Vagina (12)		12	314	2674
	
	Other tissue (29)		29	1553	6521

	Aerial (11)		11	1641	3938
	
	Oil (51)		51	1202	1902
	
		Compost	52	1607	2639
		
		Food treatment	20	368	1117
		
	Artificial (640)	Industrial	222	4997	8192
		
Other (569)		Mines	107	3836	6157
		
		Other	39	1645	2628
	
	Soil-Saline waters interf (13) (13(13)		13	2334	3989
	
	Soil-Freshwaters interfase(54) iiinterfasinterfase(54)		54	3278	5106

Unknown (200)			200	6329	10889

Hierarchical classification of environments composed of three nested levels of resolution (supertype, type and subtype), showing also the number of samples, OTUs and individual sequences in each.

**Figure 1:**
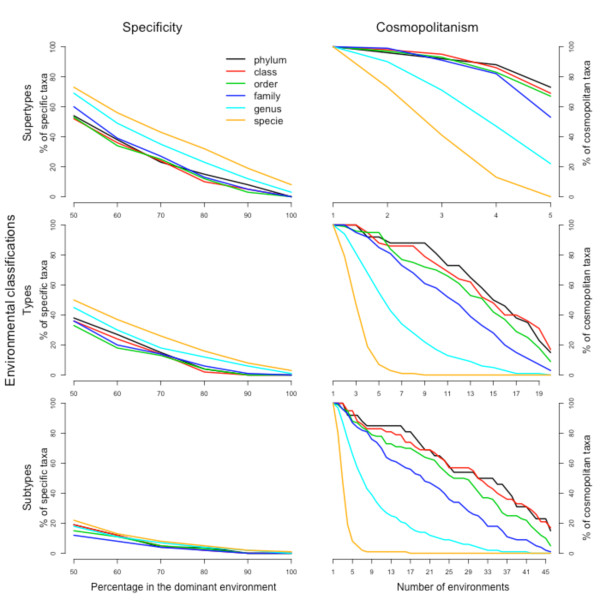
**Quantification of specific and cosmopolitan taxa**. Left side: percentage of specific taxa for the three levels of environmental classification. A particular taxa is defined as specific when a given percentage of its observations belong to a single environment. That percentage is shown in the abscissa axis. Right side: percentage of cosmopolitan taxa for the three levels of environmental classification, in relation to the number of environments in which the taxa is present.

First, we determined the abundance of each taxon in all the environments, to study the patterns of specificity and cosmopolitanism. The results are shown in Figure 1. If we define a specificity criterion as having 90% or more of their observations in a single environment (which can be very restrictive depending on the resolution of habitat classification), the apparent trend is to increase it with taxonomic depth. Higher taxonomic ranks (phylum, class, order and family) have approximately the low specificity percentages, while for genera and especially species there is a clear increase in the amount of specific taxa. Nevertheless, the percentage of specific species does not even reach 20% for the most favourable case of environment supertypes (using 90% for the specificity criterion, Figure 1). Some of these species belong to well-known examples of specificity, such as the marine bacteria *Prochlorococcus marinus *and *Pelagibacter ubique*. These taxa are thought to be amongst the most abundant microorganisms in the Earth [22], but at the same time they are specific from the pelagic marine environment: they are typical examples of specialists living on a widely extended habitat on the Earth. For these taxa, however, genetic differences that can be associated to niche differentiation have been reported, showing that specificity could be found on subspecific (ecotype) level [23]. The gastrointestinal tract of animals is, once more, the environment where more specific bacteria can be found.

It must also be remarked that for environmental subtypes, the most detailed level of the environmental classification, specificity is almost inexistent at any taxonomic depth (Figure 1). The relatively low numbers of specific bacteria, even at the species level, indicate that, using this environmental classification, environment-specific clades of bacteria are not abundant and therefore clear-cut specialization is not a widely used strategy in prokaryotes.

We can define a cosmopolitan taxa as having five or more observations in 90% of the environments (5 of 5 for supertypes, 18 of 20 for types and 41 of 46 for subtypes). While the upper taxonomic ranks can be considered as eurioic (tolerant to highly diverse conditions), that behaviour does not necessarily hold for their constituents. This trend can indeed be appreciated in Figure 1, where cosmopolitanism decreases greatly for the genus level and disappears almost completely for species. Again, the upper taxonomic levels (phylum, class and order) show a uniform behaviour, with high levels of cosmopolitanism (around 70% of the taxa for environmental supertypes, and 30% for subtypes). This trend starts to change for taxonomic families, but a sharp decrease is observed for genera and species. This observation is concordant with the parallel increment in specificity, and indicates that environmental selectivity manifests mainly at genus or species level.

**Figure 2:**
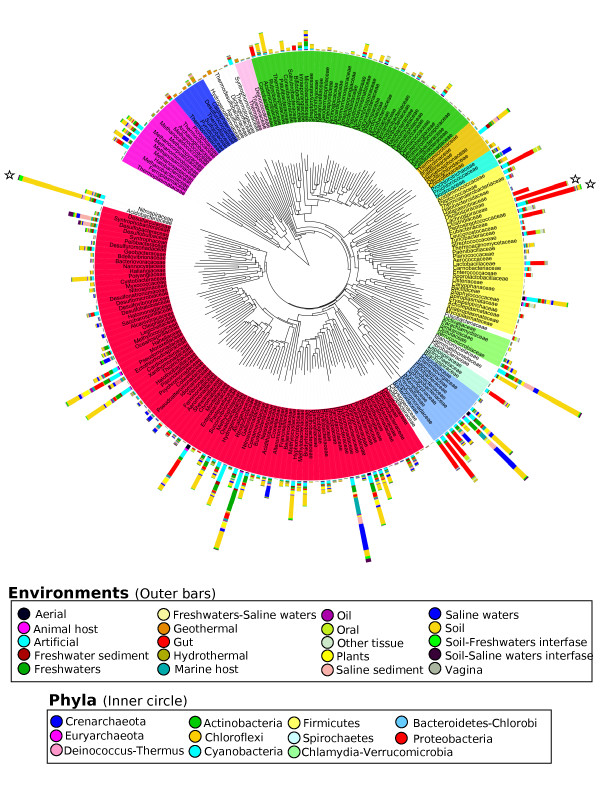
**Distribution of individual taxonomic families in the different environment types**. The phylogenetic tree shown in the inner circle was created by taking one representative sequence from each family, and was arbitrarily rooted in the branch separating bacteria from archaea. Families are coloured by its corresponding phyla, and only families with 10 or more observations have been considered. The bars in the outer circle indicate the number of times that each family has been observed in a sample from a particular environment. The bars marked with stars have been reduced to one third of their original size, for clarity purposes. This figure was done using iTOL server[42].

Focusing in families, Figure 2 illustrates their representation in the diverse environments. It is apparent that most families can be found in many different environments, with only a few presenting a clear-cut specificity. According to the specificity criterion cited above, just 3 out of the 211 families (1.4%, see Figure 2) will be specific for environmental types: two *Clostridia *(*Lachnospiraceae *and *Oscillospiraceae*), and the gamma-proteobacterial family *Succinivibrionaceae*, all of them specific for the gastro-intestinal tract of animals (Additional file 1, Table S1). These are strictly anaerobic chemoorganotrophs that are found in the rumen of cattle, sheep and other animals. The distribution of different species within these families can nevertheless be quite heterogeneous depending on the diet of the animal, according to the available carbon and energy sources [24]. When using the broader classification of environmental supertypes with the same criteria, we found specificity for 13 families (6.1%), mainly from thermal and host-associated habitats (Figure 2, and Additional file 1, Table S1). No specific families were found, however, when using the most detailed classification of environmental subtypes. Hence, we can say that under this criterion, specificity is a rare event in taxonomic families. If we relax the specificity criterion, the number of putative specific families increases, but such criteria are probably too loose and inadequate for determining specificity.

In contrast, cosmopolitanism seems to be more common for families, with their members well distributed in most environments. Two clear examples can be found in *Pseudomonadaceae *or *Flavobacteriaceae*. By defining a cosmopolitan family as having five or more observations in 90% of the environments, we found that 111, 23 and 4 families met these criteria for environmental supertypes, types and subtypes, respectively (Figure 2 and Additional file 1, Table S1). Therefore, for that taxonomic level, there is more likelihood of finding instances of cosmopolitanism than of specificity. But this could also simply be due to the lack of enough specific features within this taxonomic level, which nevertheless could appear at a deeper taxonomic resolution. For example, members of the family *Flavobacteriaceae *can colonize diverse ecological niches with a wide range of physical-chemical characteristics [25]. It is also possible that our classification is too broad, even at subtype level, to capture the possible patterns of environmental specificity.

To exclude possible biases due to unequal size of the samples, we created subsets comprising just samples of comparable size. The results of cosmopolitanism and ubiquity for two of these datasets are shown in Additional file 2, Figure S1, showing that the general trends exposed above are well conserved in these and other subsets.

Cosmopolitanism and specificity patterns can also be revealed by inspecting the evenness of the distribution of a particular taxon in the different environments. This can be done by calculating biodiversity indices. For a particular taxon, high diversity values indicate both presence in more environments and a well-balanced distribution across them, as expected for ubiquitous families, while low diversity indicates preference for some environment(s). The results (Additional file 3, Table S2) suggest that the most diverse families with respect to their environmental distribution are *Pseudomonadaceae, Comamonadaceae*, *Caulobacteraceae, Flavobacteriaceae *and *Xanthomonadaceae*, while amongst the least diverse families we find *Pyrodictiaceae, Aquificaceae *and *Nautiliaceae *(in hydrothermal environments), *Thermoactinomycetaceae *(soil), *Sulfolobaceae *(geothermal), *Oscillospiraceae *and *Lachnospiraceae *(gut).

It is apparent, however, that even in the absence of total specificity, some taxa show a marked preference for some environments. For instance, some archaeal clades have been found mostly, but not exclusively, in thermal samples. To quantify these preferences (affinities), we used a Bayesian hierarchical statistical model for detecting differences between the observed and expected distributions of abundances of the taxa in the environments, under the assumption of statistical independence between taxa and environments. The results are presented in Additional file 4, Figure S2. The highest affinities were found for taxa present in thermal environments (families *Aquificaceae*, *Sulfolobaceae*, *Thermoproteaceae *and *Thermococcaceae*), or in association with human tissues (*Pasteurellaceae *for oral, *Lactobacillaceae *for vagina, or *Oscillospiraceae *for gut). Here, 180 of the 211 families (85% of the total) show a high affinity for at least one environmental type, and 52 (25%) do for just one. This does not imply environmental specificity but does, undoubtedly, indicate a clear environmental preference.

The families that are present in many environments, but not showing relevant affinity values for any of them, may be considered ubiquitous. Amongst these we mainly find *Proteobacteria*, such as *Xanthomonadaceae*, *Comamonadaceae, Pseudomonadaceae *and *Burkholderiaceae*, but also the archaeal *Methanosarcinaceae *and the Clostridia *Peptococcaceae *families.

It is interesting to note that the *Clostridia *clade harbors cosmopolitan families, such as *Peptococcaceae*, and environment-specific ones such as *Lachnospiraceae *or *Oscillospiraceae*. This indicates that phylogenetically close families can show strikingly different environmental preferences and distribution patterns, which at least for some cases, questions the validity of the proposed relationship between phylogenetic distance and environmental preferences [26,27].

**Figure 3:**
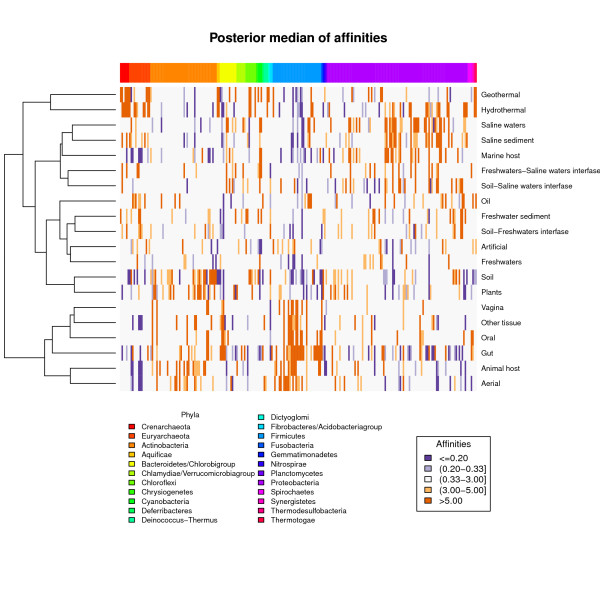
**Relations between environments, and between environments and taxonomic families**. Heat-map of the posterior medians of the affinities and the resulting dendrogram from the cluster analysis of the environment types, using log-affinities and euclidean distance. Purple and orange cells represent low and high affinity values, respectively.

Taxonomic distributions can be used to explore the characteristics of the environments themselves. Grouping environments according to similarity in their taxonomic profiles can help us to understand the main environmental features at play in selecting prokaryotic diversity. To assess the relationship between environments and taxa, we clustered the different environmental types according to the affinities of their different taxa (Figure 3).

The environments are separated into five different groups. The first one is associated with animal tissues (oral, gut, vagina, other human tissues, samples from animal tissues and aerial specimens, the last mostly coming from air expired from human subjects). These habitats clearly differ from the rest, and some of the prokaryotes living there do not thrive in other locations [28]. Thus, host association with animals emerges as the first discriminating factor in the composition of the prokaryotic assemblages.

The second group to segregate is composed of thermal environments (geo- and hydrothermal), and also shows a clearly distinct taxonomic profile. Both environments are separated by long distances in the dendrogram, which indicates significant differences between them. The absence of oxygen and light in hydrothermal locations accounts for the presence of some anaerobic methanogenic archaea in hydrothermal, but not geothermal sources, or for some photosynthetic cyanobacterial families that are located only in geothermal spots where light is present.

The third group comprises saline environments, and is represented mainly by heterogeneous marine samples which show quite similar profiles. Athalassohaline waters of saline inland lakes (including soda lakes, with a mineral composition different from marine waters) also cluster within this group, showing that salinity as a whole, and not salt composition, is the determinant ecological factor. This is related to osmotic adaptations of the organisms. The fourth group contains terrestrial samples from soil and plants. Finally, the last group is dominated by freshwater samples. It is very interesting to note that freshwater samples are more related with terrestrial samples than with marine ones. This indicates that salinity is a very important selective factor for the composition of prokaryotic communities, and more relevant than the apparently loose distinction between aquatic and terrestrial media, as was also described by Lozupone and Knight using a strictly phylogenetic approach [20]. Many prokaryotic taxa found in soil samples, may actually thrive in the interstitial water within soil particles [29], which could explain the highest similarity between the taxonomic profiles of freshwater and soil environments.

When performing the analysis for environmental subtypes, the trends above are shown again, but new details emerge (Additional file 5, Figure S3). As before, host-associated habitats obviously separate from the rest, but on this occasion the cluster includes the samples related to food treatments and compost. Thermal environments form the second clear division. The next groups to separate correspond to nutrient-rich soils (forests, grasslands and agricultural soils), and to saline environments. Interestingly, the latter are all aquatic except for saline soils, which cluster with this saline subgroup rather than with other soil subtypes, thus illustrating the importance of salinity. The remaining groups are formed by a mixture of artificial, freshwaters and nutrient-poor soils that do not separate clearly. The conspicuous distinction between rich and poor soil types correlates with the increase of several taxa in rich soils (especially *Actinobacteria*), and is in accordance with previous studies [30].

**Figure 4:**
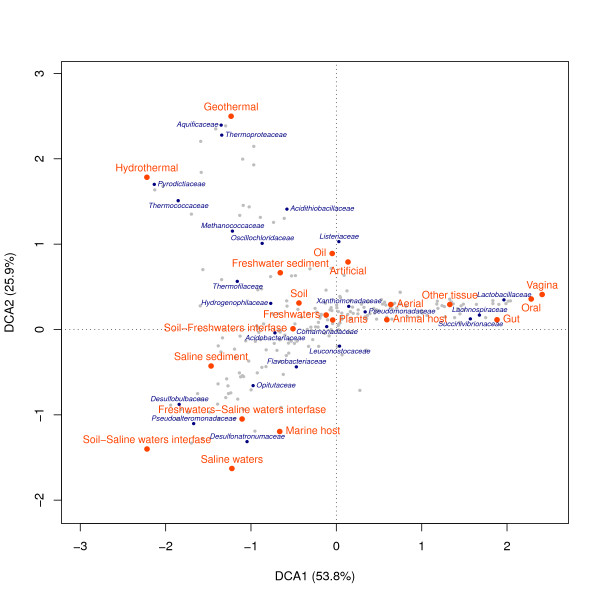
**Bi-plot of environment types and taxonomic families**. The axes correspond to the first two components of a detrended correspondence analysis (DCA). Percentages in brackets refer to the proportion of inertia explained by the axes.

To further explore the relationships between environments and taxa, we carried out a Detrended Correspondence Analysis (DCA), a well-known multivariate technique traditionally used in ecology to explore patterns of variation in community data matrices. Figure 4 shows the results for family level. The first two resulting axes allow the discrimination between environments according to their taxonomic profiles. The first axis clearly separates animal tissues from other environments. The second axis discriminates saline and thermal environments from the rest. Freshwaters and soil samples are nearby and they both are close to the origin, thus indicating the absence of very specific taxa in them. This result supports the division in the five main environmental groups found earlier.

A measure of the complexity of the composition of the different environments can be obtained by means of the diversity indices calculated from the abundance of taxa in the samples from these environments. Low diversity values for a given environment indicate that some taxa are highly prevalent (appear in most samples from that environment) and dominate, while high diversity represents less dominance and a more balanced composition. The values of the Shannon's index of diversity for the different environments are displayed in Additional file 6, Table S3, and the histograms showing the distributions can be seen in Additional file 7, Figure S4. Amongst the most diverse environments, we find artificial, freshwaters and soil. The artificial environments are very heterogeneous and sparse, and hence a high variability between samples is expected. Freshwaters and soils environments do not appear to be very restrictive, as commented above and, therefore many taxa are present and none dominates clearly. The least diverse habitats are host-associated, thermal or saline, indicating that the strong constraints imposed by these environments (such as anaerobiosis, high temperatures or high salt content) greatly limit the representation of taxa.

**Figure 5:**
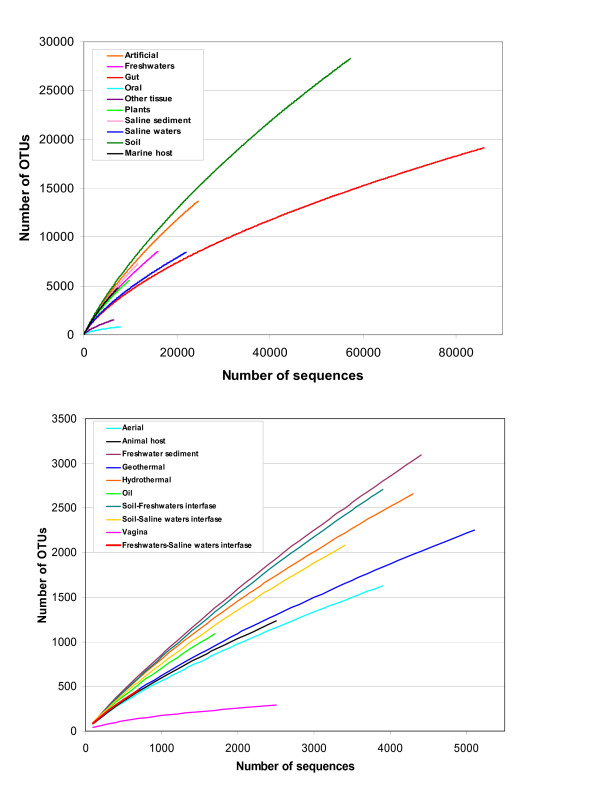
**Collector's curves**. Collector's curves for the abundance of sequences and OTUs in all the environments.

Finally, we are interested in exploring how complete our knowledge is about the richness of species in the different habitats considered in this study. By using the distribution of sequences and OTUs in the samples of a given environment, we derived a collector's curve which illustrates the rate at which new OTUs are found as more samples are sequenced. This curve indicates the present coverage of the environments and the completeness of the current knowledge about the abundance of OTUs, thus also providing a comparison of the richness of the different environments. The curves (Figure 5) show that the highest richness in OTUs can be expected for soil, freshwater and artificial environments, while saline waters and all thermal and host-associated environments appear as less rich. This is in good agreement with our previous results. Nevertheless, the pyrosequencing of individual marine samples have determined that saline waters are very rich in species [31]. That observation is not in contradiction with our results, because here we consider sets of samples, not just individual ones. Individual marine samples can be richer than samples from other environments, especially if they have been exhaustively sequenced. But it is also likely that other environments can harbour more species than sea waters [32], which can be related to the variety of different niches.

It is also important to notice that most curves show no saturation (i.e., they are far from reaching their respective top plateaus). Therefore, we can conclude that there is still a long way to obtain a complete description of species diversity for almost any environment. The only exceptions may be human tissues (vagina, oral and other tissues) where their respective curves show a relative saturation, thus indicating that we have already observed the majority of the putative species in these habitats. The curve for gut does not show this clear saturation, and one reason is that it includes samples from very different animals (not only humans), thus making it more heterogeneous than the samples from the rest of human tissues.

## Discussion and Conclusions

In short, our results indicate that most taxa can be found in many different environment types. Environmental specificity is not very common, although clear environmental preferences exist. The most selective environments, where more specialist taxa can be found, are animal tissues and thermal locations. Salinity also emerges as a very important factor in shaping prokaryotic diversity. These results are in accordance to previously described patterns [20]. The specificity of their characteristic microbial inhabitants is then better explained by the adaptations of these microorganisms to the environmental constraints than by geographic isolation of these habitats. In contrast, soil and freshwater habitats are the least restrictive environments as they harbor the highest number of prokaryotic taxa and species. This is probably related to the heterogeneity of these environments, in which, besides a relative homogeneity for some ecological factors, a wide range of physical-chemical and biotic factors can be found and, therefore, many different niches are available, thus being suitable to be colonised by a variety of prokaryotic taxa. For instance, although it could be though that freshwater habitats are relatively homogeneous, strong environmental gradients are found within freshwater bodies (see [33], for multiple examples). In the samples considered in our study, a broad variety of environmental features are represented for freshwater habitats, such as for trophic status (from oligotrophic to hypereutrophic), limnological features (e.g shallow mixed to deep stratified lakes), and others.

Nevertheless, some caveats of this study must be taken into account. It is necessary to consider whether the patterns of taxa distribution in those environments are linked to either environmental factors or to historical events bound to habitat isolation [6]. Many taxa have been found in particular environments only occasionally, which could indicate that they might not be active members of the communities thriving in these locations. Indeed for soil environments, it has been proposed that many of the species found in a particular location are inactive [34]. The bacteria capable of sporulating are clear candidates for such a role, as has also been observed for microbial eukaryotes in freshwater sediments [2]. For instance, spore-forming genus *Bacillus *is the second most abundant genus in this dataset, only after *Pseudomonas*. This could be a general case for prokaryotic communities, which may be composed of "core members" (as indicated by the high similarity found between samples from the same environments, see Additional file 8, Figure S5), and sporadic members which are limited by niche specificity and competitive interactions, and which remain mostly inactive until a change in the media conditions favours their growth. They could also correspond to transient species, which are accidentally passing, although a recent metagenomic analysis found a very low rate of sequences from putative transient species [35].

We found that most OTUs have been observed once (Additional file 9, Table S4). We have deliberately omitted these OTUs from the analyses of cosmopolitanism and specificity, because their low abundance does not allow to extract conclusions about their environmental distributions. Nevertheless, their inclusion does not affect significantly the conclusions extracted for all taxonomic ranks, except that of species (Additional file 10, Figure S6). Further study is required to understand why the majority of OTUs are rare, and some work has already been done by Sogin and colleagues to address this point [31]. As commented above, they could correspond to specialist species with a very limited niche. But it is also likely that the limited size of samplings cannot recover low-abundance OTUs from the environments and samples where they actually exist. After all, it is virtually impossible to conclusively show that a microbial taxon is absent from a given location by the current sequencing methods [6].

Also the heterogeneous size of the samples can introduce a bias in the results, because big samples are likely to recover more species than small ones. Also rare OTUs are more likely to be detected in larger samples. Information about the abundance of each taxa in each sample could provide relevant information to correct this size effect. But unfortunately, this information is not present in the original source of data. Therefore, the patterns described here could be affected because samples of different size are being considered. To exclude this possibility, we created smaller datasets composed uniquely of samples of comparable size. The results of cosmopolitanism and ubiquity for two such datasets are shown in Additional file 2, Figure S1. It can be seen that the patterns are very similar to the ones obtained with the full dataset. Also in the correspondence analysis we transformed the data dividing frequencies by the number of samples instead, as a proxy for the number of sequences, thus assuming that larger samples tend to have more sequences. Finally, in the Bayesian model of affinities, we included random effects to partially account for the variation of the unknown number of sequences.

It is also necessary to consider that most data have been obtained by the standard sequencing procedures which involve PCR amplification steps using "universal" primers, a procedure that is known to be biased [36,37]. Universal primers are designed according to current knowledge and could perform poorly or even miss species or taxa that remain unknown. Another source of potential biases is that in clone library sampling, often just some few clones of interest are sequenced or submitted, discarding the rest. To evaluate community composition, we need new and more accurate approaches that do not rely on primers or probes. Nowadays, new sequencing technologies can provide the adequate framework for the unrestricted sequencing of 16S rRNA gene sequences or of other universally conserved genes [36] that can be used to accurately describe prokaryotic diversity. It is expected that the samples analysed in this way can describe better the real diversity and to unveil the presence of specialist species.

An interesting point that has not been addressed in our study is the consideration of the temporal dimension. Indeed, some of the samples have been taken in the same spots, in different sampling experiments performed at different times. A good example are the samples collected in lakes: in our dataset, there are six samples taken in Mono Lake (United States), five in Lake Cadagno (Switzerland), and four in Lake Kinneret (Israel), which differ among sampling times. Therefore, it would be possible to address the temporal variation of the microbial composition in these sites. But it is very difficult to discriminate between temporal and spatial factors. In this particular case, all these lakes display different types of vertical stratification, and the microbial communities found at different depths could vary and be influenced by the mixing regime. A temporal analysis should therefore be performed with sets of samples where all environmental features have been well characterized. And also, as above, the heterogeneous sizes of the samples and the existence of different niches can be misleading and complicate the analysis.

As far as we know, this is the most comprehensive assessment of the distribution and diversity of prokaryotic taxa and their associations with different environments. We expect that this and further studies can help to gain a better understanding of the complex factors influencing the structure of the prokaryotic communities.

## Methods

### Obtaining sequences and grouping in samples

We collected 16S rRNA gene sequences from the environmental section of GenBank database, comprising the results of many different 16S rRNA sampling experiments. After discarding short (less than 250 bps) and long (more than 1900 bps) entries, we have obtained a data set of 399.098 16S sequences of variable length from bacterial and archaeal species. Each sampling experiment is identified by its reference (title of the study and authors), and the individual sequences are assigned to their original sample. A total of 4.334 samples were identified, that reduced to 3.502 when we eliminated those with less than five sequences. It is important to notice that the original source can describe each sample exhaustively, listing each sequence found, or rather enumerate just the different genotypes by removing the identical sequences. The second case is the most common one, in which no information about the abundance of individual genotypes is present. In these instances, we can obtain information about the richness (number of genotypes) of each individual sample, but not about its diversity (abundance of each genotype). For normalizing the minority of cases in which some of this information is present, identical sequences were eliminated by using cd-hit [38] with identity parameter set to 100%, producing a final data set containing 359.928 sequences.

### Classifying samples in environmental categories and environmental features

We have derived a classification of environments to categorize the collection of samples. The environments are classified in 5 supertypes, 20 types and 46 subtypes, as can be seen in the schema shown in Table 1.

We have used a semi-automatical text-mining procedure for classifying the samples in these environmental categories [39]. The performance of the classifier is fairly good, producing results for 52% of the samples with a precision of 81%. The results were checked by human experts, correcting the possible mistakes and increasing the coverage by annotating unclassified instances. By this procedure, 3.181 samples (91% of all samples) were classified (Table 1).

In some instances, a single sample is composed by different individual sampling experiments, which have been merged for submission to the database. Usually this is not an obstacle for classification and for the final objective of describing taxonomic diversity of the different environments, because all individual samples come from the same or very similar environments (different rivers, different guts of termites, different water treatment plants, etc). In the few instances (43 samples, around 1% of the total) in which the individual samples come from diverse environments (for example, a river, its estuary, and the adjacent ocean), they have been classified in all of these environments, thus reflecting the multiple origins of the sequences. The results were unaltered when we repeated the analyses excluding these 43 samples.

### Identifying OTUs

We have grouped closely related sequences into OTUs using cd-hit [38], clustering sequences at 97% identity, which is often proposed as a reference level that may separate different prokaryotic species [17]. This resulted in 124.390 different clusters, which were considered as OTUs. 67% of these OTUs are composed by a single sequence (Additional file 9, Table S4), and were excluded for the study of specificity and cosmopolitanism.

### Taxonomic assignment of sequences and OTUs

Each of the sequences was assigned to a reference taxon by using RDP classifier [40], considering only the assignments with more than 80% confidence. This resulted in predictions for 356.250 sequences, corresponding to different taxonomic ranks. Additionally, we also used an assignment procedure based on Blastn searches against Greengenes database http://greengenes.lbl.gov, collecting the bit-scores for the five best hits belonging to each taxa, and finding the taxa with the best average score and a fixed difference to the second best. This procedure assigns 90% of the sequences to phyla, 81% to classes, 72% to orders, 65% to families and 47% to genera. At family level, 85% of the assignments are coincident between both approaches.

OTUs were classified by extracting a consensus from the taxonomic assignments of their individual sequences. The objective was to find the taxon that dominates at the lowest possible taxonomic rank, fulfilling the following criteria: having more than five sequences in the OTU, and being the only taxon with at least 25% of the sequences of the OTU assigned to it. The usage of either RDP or Greengenes assignments produced coincident assignments for 91% of the instances, and does not alter the results significantly. Unless stated otherwise, the results shown correspond to RDP assignments.

### Collector's curves

To create collector's curves for the distribution of OTUs in environments, a single metasample was created for each environment, pooling together all the sequences from the samples corresponding to it. We simulated the sampling of the metasample by picking up individual sequences randomly, with non-replacement. To produce the curve, we checked whether another sequence for the corresponding OTU had already been seen or not. The simulated sampling continued until no sequences were left. The full procedure was repeated ten times, and the individual curves were averaged to obtain a final result.

### Statistical analyses

We computed a two-way table with the number of different OTUs per taxa and environment. To assess the level of bacterial biodiversity of the different environment types and the degree of ubiquity of the taxa considered, we computed Hill biodiversity numbers [41] using this abundance community matrix for both taxa and environments, respectively. We considered Hill numbers for the scale values 0, 1 and 2 which, for a given environment, for example, correspond to the total number of families, the exponential of the Shannon index of biodiversity, and the inverse Simpson index.

Exploratory data analyses revealed that those environments with more samples tended to have more OTUs. To remove this 'size' effect, we transformed the data by dividing the frequencies in each column by the number of samples in that environment, thus creating a community matrix which contained the average number of OTUs per sample for each taxa and environment type. We then carried out a Detrended Correspondence Analysis (DCA) to explore the variation in the transformed abundance matrix.

We also fitted a Bayesian hierarchical model to the original community matrix in order to quantify the affinity between taxa and environments. In the first layer, our model assumes a Poisson distribution for the number of OTUs Y_ij _observed in the taxonomic family i and environment type j. The mean of this distribution is assumed to be λ_ij_E_ij_, where E_ij _is the expected number of OTUs under the hypothesis of independence between the distributions of taxa and environments, and λ_ij _is the under- or over-presence of family i in environment j. The values of λ_ij _> 1 indicate the affinity of the family for the environment, whereas the values of λ_ij _< 1 suggest a lack of affinity. In the second layer, the 'affinities' λ_ij _(on the log scale) are decomposed into the taxa and environment main effects plus an interaction: log λ_ij _= α + θ_i _+ γ_j _+ ν_ij_. The main effects of taxa and environments can be interpreted as surrogates for the unobserved variables that associate to each one. The interaction terms (or residuals) can be seen as an adjusted affinity, that is, the part of the over- or under-presence that cannot be accounted for by the factors linked to the taxa or environment.

Statistical inference was performed under the Bayesian paradigm, which implies assigning prior distributions to the parameters. We chose normal distributions for each of the main effects and a mixture of two normal distributions for the interactions. One of the components of the mixture is intended to pick up noise, whereas the other aims to pick up true departures from the main effects. We implemented the model in JAGS http://mcmc-jags.sourceforge.net, a free-license software for Bayesian inference. The outputs from this analysis were samples from the posterior distribution of the model parameters. We then represented the posterior median of the affinities between taxa and environments using a heatmap; we chose a dichromatic scale from purples to oranges. The former represent low affinity values (meaning an underpresence of the taxa in the environment), whereas the latter represent affinity (overpresence). We used standard hierarchical clustering with Euclidean distance to group the environment types according to the values of their taxa affinities (on the log scale). The resulting cluster dendrogram is displayed next to the heatmap to make visualization and the interpretation of the results easier.

### Database creation

We have created envDB, a mySQL database containing all the data associated with this work. The user can perform queries on sequences, OTUs, samples and environments under a flexible and user-friendly interface. The database will be updated regularly and its capabilities are described elsewhere [39]. The database is available at http://metagenomics.uv.es/envDB

## Authors' contributions

JT and AM conceived the study. JT and JJA designed the methods. JJA performed all statistics. MP created the database. JT, JJA and AC analyzed the results and extracted the conclusions. All authors drafted, read and approved the manuscript.

## Supplementary Material

Additional file 1 **Table S1**. Dominant environments for taxonomic families. Click here for file

Additional file 2 **Figure S1**. Specificity and cosmopolitanism plots (see figure 1) for a limited set of data, composed of samples with approximately the same number of sequences. Upper: samples having between 10 and 20 sequences each (836 samples). Lower: samples having between 10 and 30 sequences each (1300 samples). Only the results for the "type" level in the environmental classification are shown. Click here for file

Additional file 3 **Table S2**. Biodiversity indices for taxonomic families. Click here for file

Additional file 4 **Figure S2**. Affinities of the taxonomic families for the different environment types, depicted using the same diagram as figure 2. The bars in the outer circle indicate the affinity of each family for the particular environments, calculated as described in the text. This figure was done using iTOL server[42]. Click here for file

Additional file 5 **Figure S3**. Heat-map showing the relationships between environment sub-types and with taxa. Click here for file

Additional file 6 **Table S3**. Biodiversity indices for environments. Click here for file

Additional file 7 **Figure S4**. Diversity plots showing the taxa ranked by their presence in the samples from each environment. The distributions are used to calculate diversity according to Shannon's index. Click here for file

Additional file 8 **Figure S5**. The first two components of a DCA of the experiments-taxa community matrix. Click here for file

Additional file 9 **Table S4**. Distribution of the number of OTUs in the clusters. Click here for file

Additional file 10 **Figure S6**. Specificity and cosmopolitanism plots (see figure 1), including also these OTUs that were found in just one sample. It can be seen that the trends are not very different to these shown in figure 1, with the exception of the curves for species. Since all these OTUs are considered environment-specific by definition, specificity percentage increases very much for species, and cosmopolitanism decreases in the same way. Click here for file
