# Supplementary material for: Environmental distribution of prokaryotic taxa
Source: BMC Microbiol. 2010 Mar 22;10:85. doi: 10.1186/1471-2180-10-85 (PMC2850351; doi:10.1186/1471-2180-10-85)
Supplement: Additional file 2 — Figure S1. Specificity and cosmopolitanism plots (see figure 1) for a limited set of data, composed of samples with approximately the same number of sequences. Upper: samples having between 10 and 20 sequences each (836 samples). Lower: samples having between 10 and 30 sequences each (1300 samples). Only the results for the "type" level in the environmental classification are shown. [file 1471-2180-10-85-S2.PDF]

Types

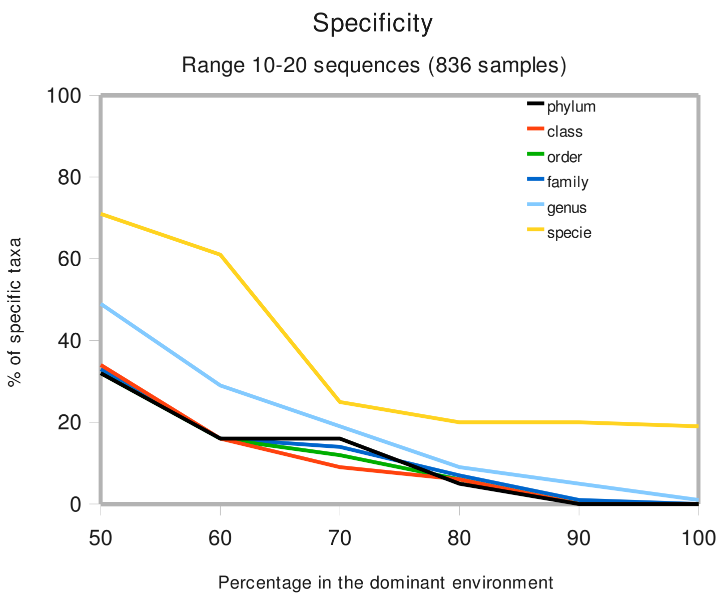
Specificity
Range 10-20 sequences (836 samples)
% of specific taxa
Percentage in the dominant environment
phylum
class
order
family
genus
specie
0
20
40
60
80
100
50
60
70
80
90
100

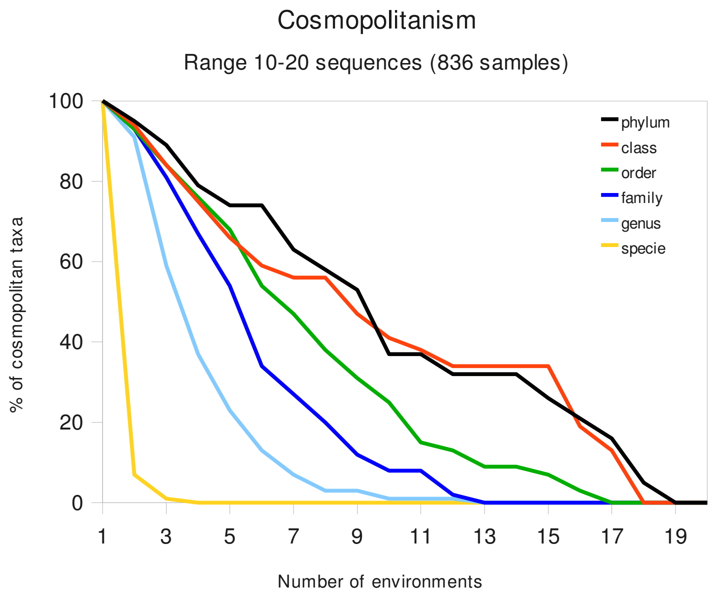
Cosmopolitanism
Range 10-20 sequences (836 samples)
% of cosmopolitan taxa
Number of environments
phylum
class
order
family
genus
specie
0
20
40
60
80
100
1
3
5
7
9
11
13
15
17
19

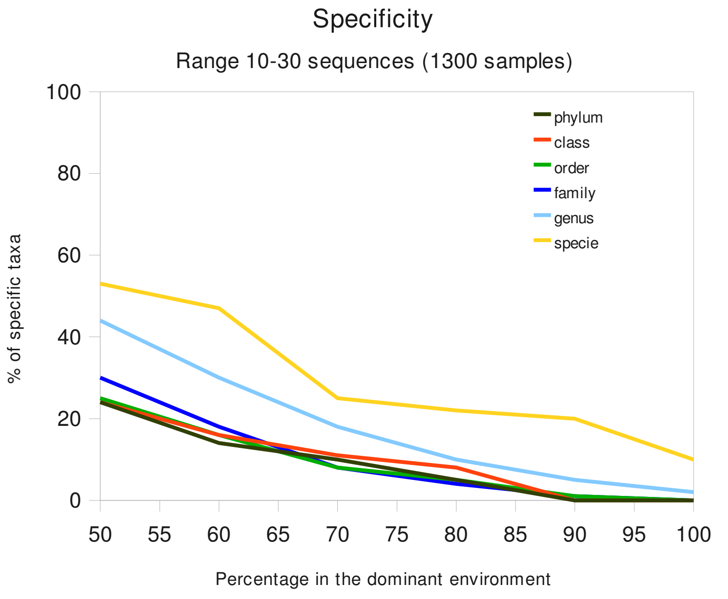
Specificity
Range 10-30 sequences (1300 samples)
% of specific taxa
Percentage in the dominant environment
phylum
class
order
family
genus
specie
0
20
40
60
80
100
50
55
60
65
70
75
80
85
90
95
100

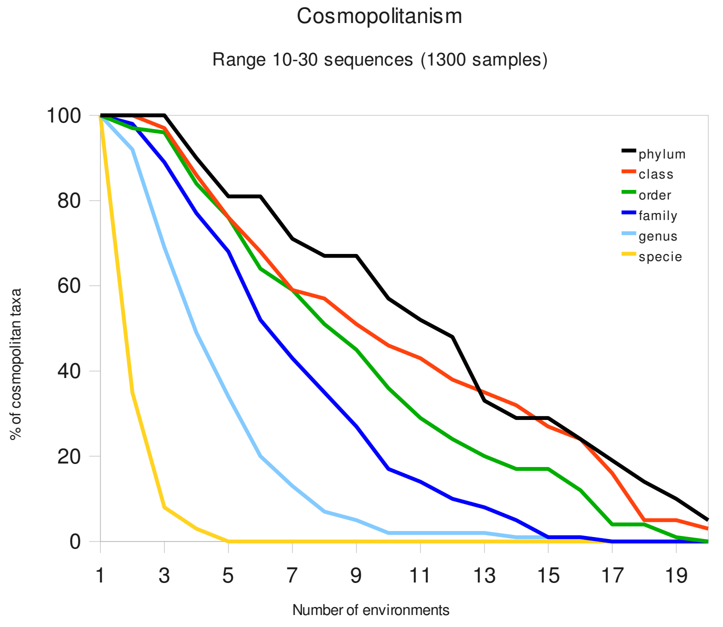
Cosmopolitanism
Range 10-30 sequences (1300 samples)
% of cosmopolitan taxa
Number of environments
phylum
class
order
family
genus
specie
0
20
40
60
80
100
1
3
5
7
9
11
13
15
17
19
