# Supplementary figures and images for: Environmental distribution of prokaryotic taxa
Source: BMC Microbiol. 2010 Mar 22;10:85. doi: 10.1186/1471-2180-10-85 (PMC2850351; doi:10.1186/1471-2180-10-85)

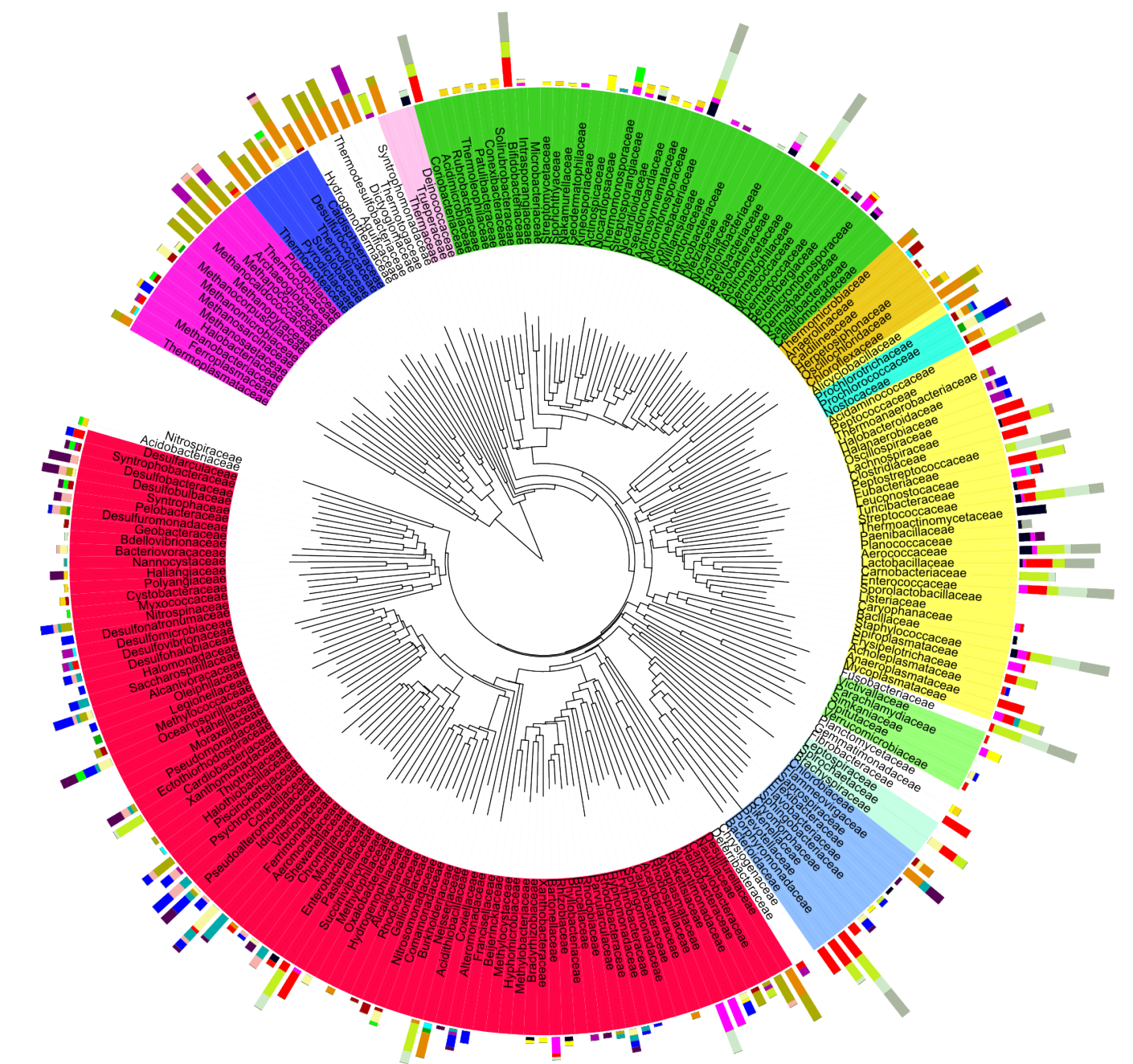

## Environments (Outer bars)

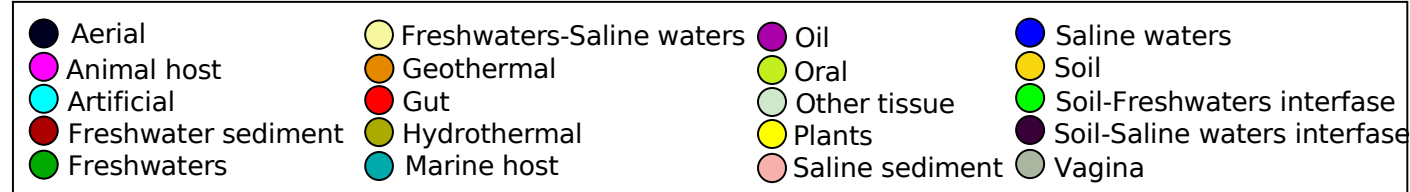

## Phyla (Inner circle)

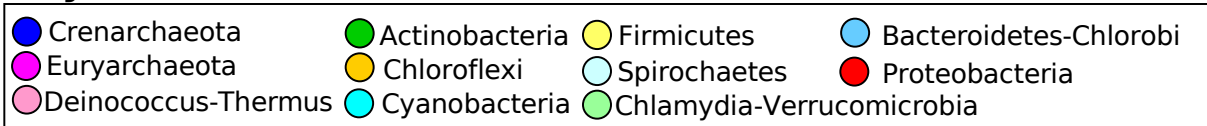

Supplement: Additional file 4 — Figure S2. Affinities of the taxonomic families for the different environment types, depicted using the same diagram as figure 2. The bars in the outer circle indicate the affinity of each family for the particular environments, calculated as described in the text. This figure was done using iTOL server[42]. [file 1471-2180-10-85-S4.PDF]

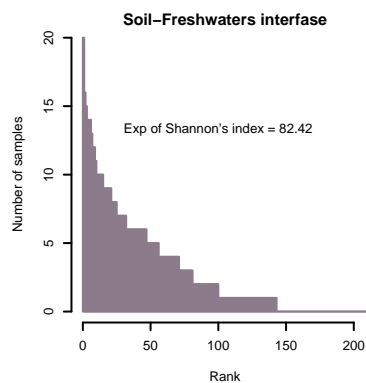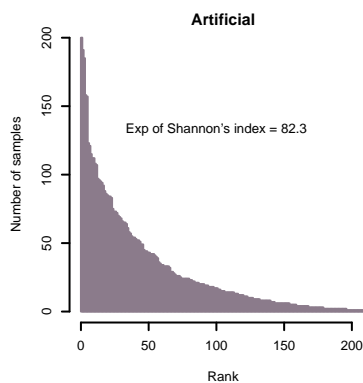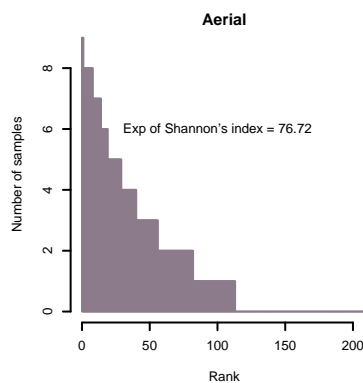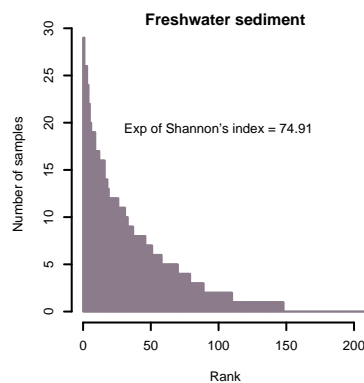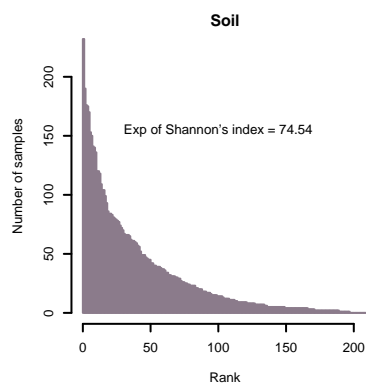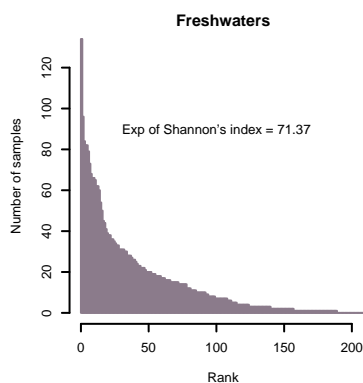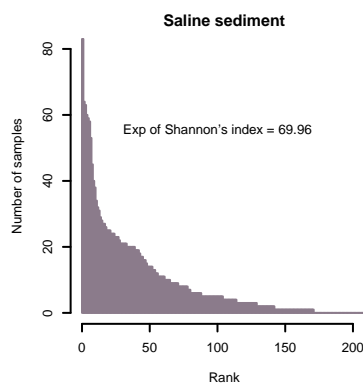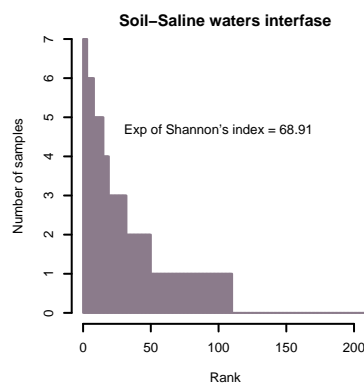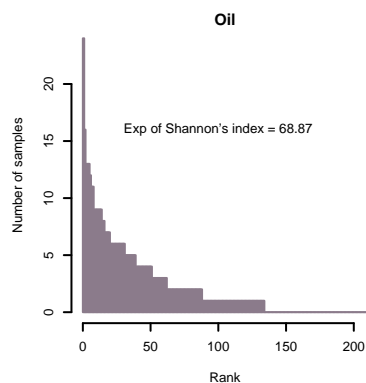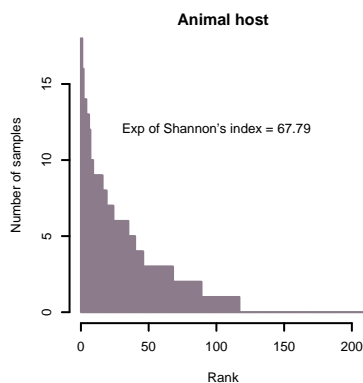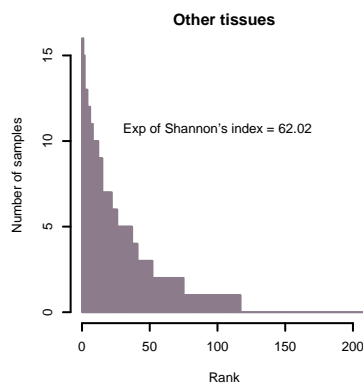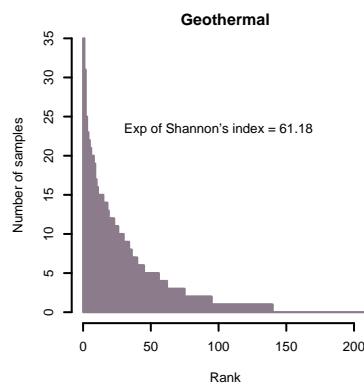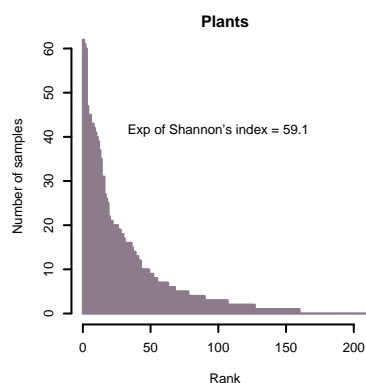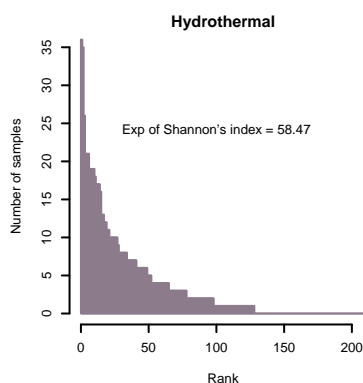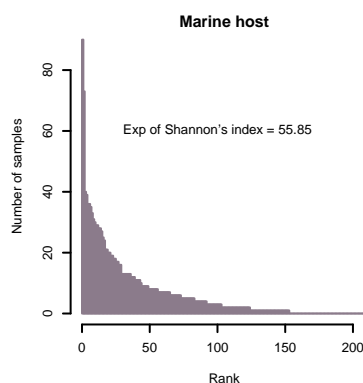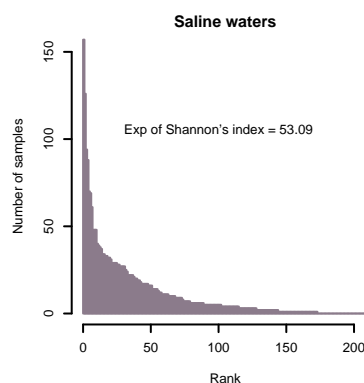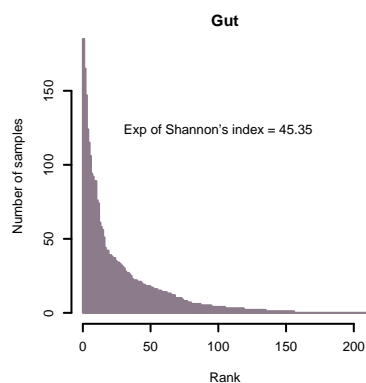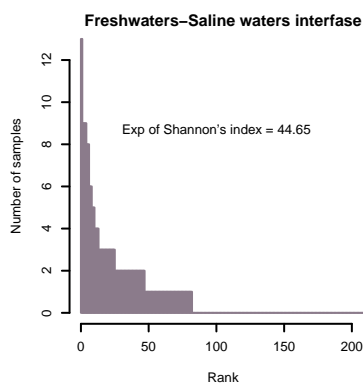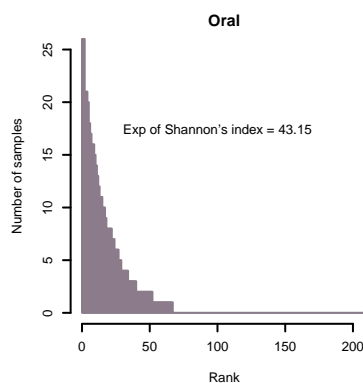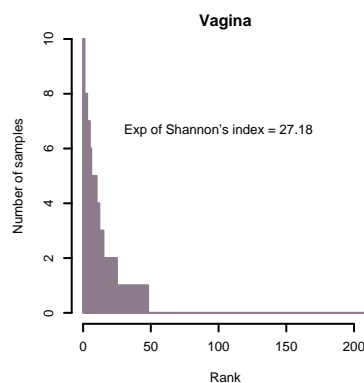

Supplement: Additional file 7 — Figure S4. Diversity plots showing the taxa ranked by their presence in the samples from each environment. The distributions are used to calculate diversity according to Shannon's index. [file 1471-2180-10-85-S7.PDF]

### Supertypes

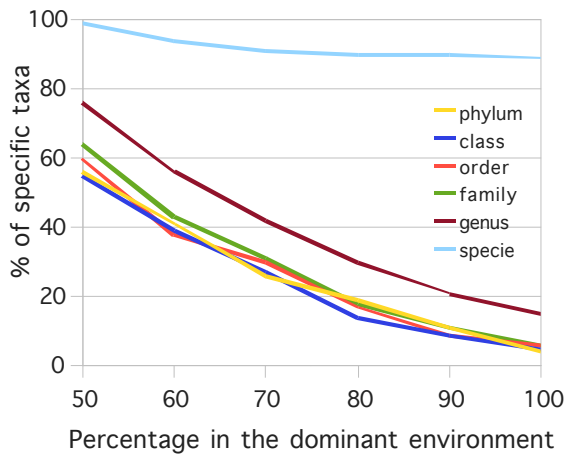

### Supertype

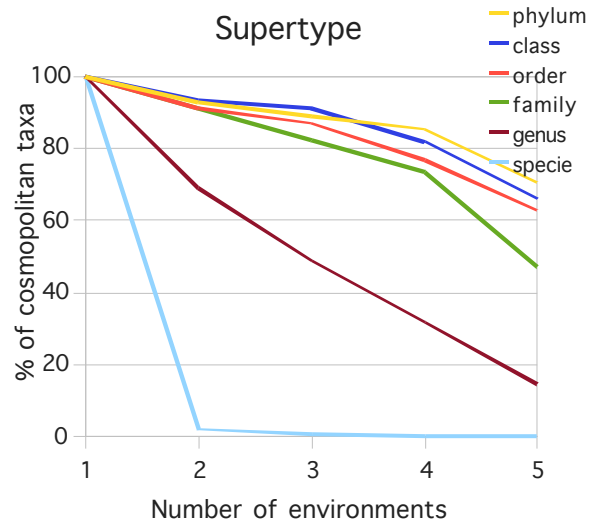

### Types

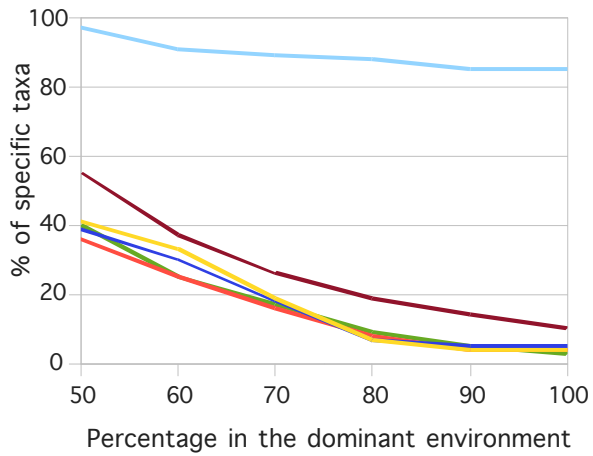

### Types

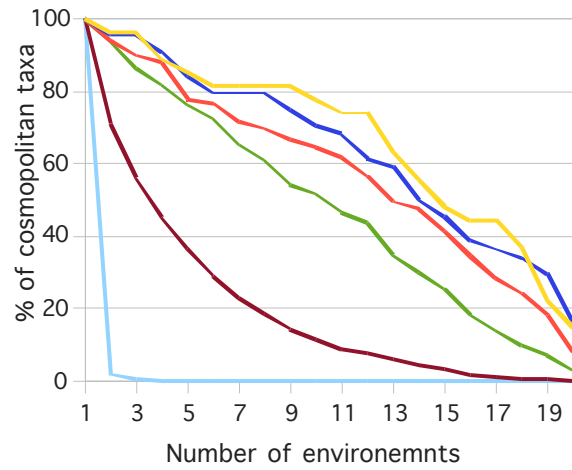

### Subtypes

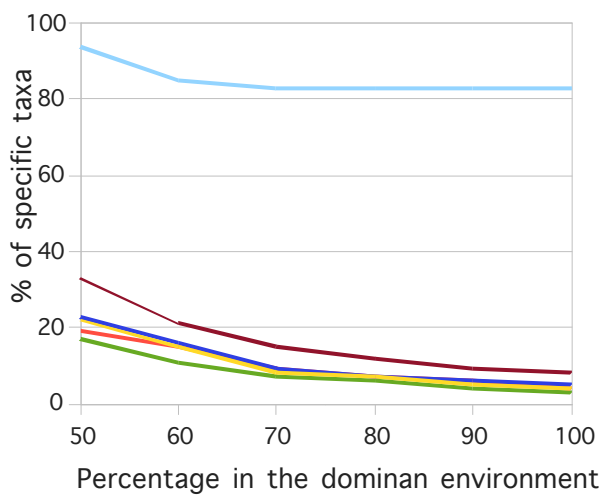

### Subtypes

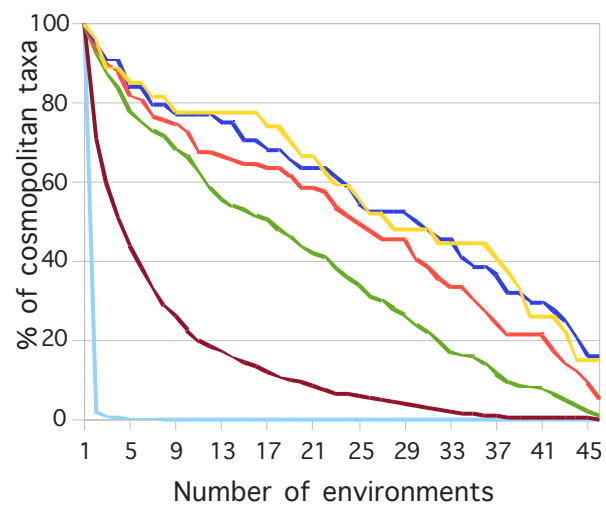

Supplement: Additional file 10 — Figure S6. Specificity and cosmopolitanism plots (see figure 1), including also these OTUs that were found in just one sample. It can be seen that the trends are not very different to these shown in figure 1, with the exception of the curves for species. Since all these OTUs are considered environment-specific by definition, specificity percentage increases very much for species, and cosmopolitanism decreases in the same way. [file 1471-2180-10-85-S10.PDF]
